# The Generation of Human Induced Pluripotent Stem Cells from Blood Cells: An Efficient Protocol Using Serial Plating of Reprogrammed Cells by Centrifugation

**DOI:** 10.1155/2016/1329459

**Published:** 2016-08-04

**Authors:** Youngkyun Kim, Yeri Alice Rim, Hyoju Yi, Narae Park, Sung-Hwan Park, Ji Hyeon Ju

**Affiliations:** ^1^Division of Cardiology, Department of Medicine, Institute for Stem Cell Biology and Regenerative Medicine and Stanford Cardiovascular Institute, Stanford University School of Medicine, Stanford, CA 94305, USA; ^2^CiSTEM Laboratory, Convergent Research Consortium for Immunologic Disease, Seoul St. Mary's Hospital, College of Medicine, Catholic University of Korea, Seoul 137-701, Republic of Korea; ^3^Division of Rheumatology, Department of Internal Medicine, Seoul St. Mary's Hospital, Institute of Medical Science, College of Medicine, Catholic University of Korea, Seoul 137-701, Republic of Korea

## Abstract

Human induced pluripotent stem cells (hiPSCs) have demonstrated great potential for differentiation into diverse tissues. We report a straightforward and highly efficient method for the generation of iPSCs from PBMCs. By plating the cells serially to a newly coated plate by centrifugation, this protocol provides multiple healthy iPSC colonies even from a small number of PBMCs. The generated iPSCs expressed pluripotent markers and differentiated into all three germ layer lineages. The protocol can also be used with umbilical cord blood mononuclear cells (CBMCs). In this study, we present a simple and efficient protocol that improved the yield of iPSCs from floating cells such as PBMCs and CBMCs by serial plating and centrifugation.

## 1. Introduction

Induced pluripotent stem cells (iPSCs) were first generated from human adult somatic cells in 2007 by Yamanaka's group [1]. Unlike embryonic stem cells (ESCs), the production and use of iPSCs are not under ethical debate. Moreover, these cells are not associated with immune rejection. Therefore, iPSCs can be useful in disease modeling, drug screening, and regenerative therapies. Patient-specific iPSCs are also promising for personalized medicine. Given these benefits, researchers are seeking to improve protocols and reach purer and higher yield of reprogrammed cells.

Most of the protocols available for generating iPSCs are currently optimized for adherent cells, such as fibroblasts [2]. Early iPSC lines were generated from skin fibroblasts obtained from patients by invasive biopsy procedures. In addition, these cells must be cultured and expanded for several passages before use in the reprogramming process.

Blood can be an ideal cell source since extraction is minimally invasive compared to skin fibroblasts [3]. However, the reprogramming of floating blood cells is relatively difficult [4]. Nucleated cells in peripheral blood consist of granulocytes including neutrophils, monocytes, T lymphocytes, B lymphocytes, and progenitor cells. Various approaches to isolate, amplify, and reprogram each cell type were reported [5–7]. However, since primary granulocytes, monocytes, and B lymphocytes are difficult to expand, they are among the most difficult cells to reprogram. In addition, in the case of B lymphocytes, it cannot be applied to clinical-grade uses because it needs an additional immortalized process with Epstein-Barr virus [8, 9]. The reprogramming of CD34+ cells into iPSCs is more efficient but involves the difficult, time-consuming isolation process of CD34+ cells from peripheral blood using granulocyte colony-stimulating factor (G-CSF) [10, 11]. Furthermore, despite several successful methods, the reprogramming efficiency of blood cells is reported to be under 0.01%, which is much lower than the yield of fibroblast-derived iPSCs [11]. Therefore, the ability to reprogram the entire cellular fraction of whole blood without having to isolate or expand any particular cell type would be an ideal approach.

Taken together, we developed a protocol to generate hiPSCs without any isolation or expansion process of a specific cell type from blood cells. Instead, transduced blood cells were serially seeded to a vitronectin-coated plate by centrifugation. This method reduced the time for attachment of reprogrammed cells and reduced the loss of reprogrammed cells. The protocol successfully worked on cord blood mononuclear cells (CBMCs) as well. Using this protocol, iPSCs were conveniently generated with high yield. Cells generated by our protocol can be further used in various fields including clinical uses.

## 2. Materials and Methods

### 2.1. Cell Culture

#### 2.1.1. Peripheral Blood Mononuclear Cell Isolation

Blood was collected into heparin-coated tubes. Collected blood was diluted with phosphate buffered saline (PBS) and centrifuged through a Ficoll gradient (Catalogue number, 17-1440-03, GE Healthcare, Little Chalfont, Buckinghamshire, UK) for 30 minutes at 850 ×g. PBMCs were collected, transferred to a new tube, washed, and resuspended in StemSpan medium (9805, STEMCELL Technologies, Vancouver, British Columbia, Canada) supplemented with CC110 cytokine cocktail (8697, STEMCELL Technologies). Cells were maintained for 5 days at 5% CO_2_, at 37°C, before use.

#### 2.1.2. iPSC Induction Using Sendai Virus

PBMCs were counted (3 × 10^5^ per well) and suspended in fresh StemSpan medium. Sendai viral particle factors from CytoTune-iPS Sendai Reprogramming Kit (A16518, Life Technologies, Carlsbad, CA, USA) were added based on the manufacturer's recommendations (3 × 10^5^ cell infectious units (CIU) of each particle per well, multiplicity of infection (MOI) = 7.5) [12]. Cells were centrifuged at 1,160 ×g, at 35°C, for 30 minutes. The cells were then incubated overnight in 5% CO_2_ at 37°C. The next day, cells were transferred onto a vitronectin (A14700, Life Technologies) coated 12-well plate and settled by centrifugation for 10 minutes at 1,160 ×g. After centrifugation, TeSR-E8 medium (5940, STEMCELL Technologies) was added. To maintain reprogrammed cells, TeSR-E8 medium was changed daily in vitronectin-coated dishes.

### 2.2. Immunocytochemical Staining

iPSCs were fixed in 4% paraformaldehyde and stained with the following antibodies: Oct4 (1/100 dilution, SC-9081, Santa Cruz, CA, USA), KLF4 (1/250 dilution, Ab151733, Abcam, Cambridge, UK), Sox2 (1/100 dilution, 630801, BioLegend, San Diego, CA, USA), TRA-1-60 (1/200 dilution, MAB4360, Millipore, Billerica, Massachusetts, USA), TRA-1-81 (1/100 dilution, MAB4381, Millipore), and SSEA-4 (1/200 dilution, MAB4304, Millipore). Alexa Fluor 594- (1/400 dilution, A11037, Life Technologies) and 488-conjugated secondary antibodies (1/400 dilution, A11029, Life Technologies) were used and cells were detected by immunofluorescence microscopy.

### 2.3. Polymerase Chain Reaction

Total mRNA was extracted using Trizol (15596, Life Technologies). RevertAid*™* First Strand cDNA Synthesis kit (K1622, Thermo Scientific, Waltham, MA, USA) was used to synthesis cDNA. Reverse transcriptase-polymerase chain reaction (RT-PCR) was conducted. Primer sequences are provided in Table 1.

### 2.4. Karyotyping

Thirty microliters of chromosome resolution additive (Genial Genetic Solutions Ltd., Runcorn, UK) was added to each well. After incubation, Colcemid® was added for 30 minutes. Cells were harvested and treated with prewarmed hypotonic solution (KCl). Fixation was performed with a 1 : 3 acetic acid : methanol solution, and slides were prepared for chromosome analysis using the trypsin-Giemsa banding technique.

### 2.5. Alkaline Phosphatase Staining

Alkaline phosphatase was purchased from Millipore (SCR004). Cells were plated and cultured for 7 days. Staining reagents used in this experiment were included in the kit. Cells were washed with phosphate buffered saline with 0.05% Tween-20 (T1027, Biosesang, Seongnam, Korea) and fixed with 4% paraformaldehyde. Reagents, including Fast Red Violet, Naphthol AS-BI phosphate solution, and water, were mixed in a 2 : 1 : 1 ratio. Staining solution was added to each well and incubated in the dark at room temperature for 15 minutes. Cells were then washed with PBST and covered with PBS to prevent drying. Stained colonies were surveyed under the microscope.

### 2.6. Functional Identification of iPSCs

To assess differentiation, the “Human Pluripotent Stem Cell Functional Identification” kit was purchased from R&D systems (SC027). Culture dishes were coated with Cultrex PathClear BME (3433-005-01, R&D, Minneapolis, MN, USA) followed by the protocol provided. After 1-2 hours, cells were prepared and seeded onto the BME-coated wells. Specific medium was prepared for each germ layer, and cells were cultured individually. After differentiation, cells were washed twice in PBS and fixed with 4% paraformaldehyde for 20 minutes at room temperature. Then, the cells were washed with 1% BSA in PBS for 5 minutes. Permeabilization and blocking were conducted with 0.3% Triton X-100 and 1% BSA in PBS for 45 minutes. Antibody against Otx2 (1/10 dilution, ectoderm), Brachyury (1/10 dilution, mesoderm), and Sox17 (1/10 dilution, endoderm) were diluted following the manufacturer's instruction and added to the cells. Used antibodies for this experiment were provided from the kit. Cells were incubated for 3 hours at room temperature. After washing, alexa fluor 568 donkey anti-goat secondary antibody (1/200 dilution, A11057, R&D) was incubated for 1 hour. Additional washing followed, and cells were covered with PBS. Staining was visualized with a fluorescence microscope.

## 3. Results

### 3.1. Establishment of iPSC Generation Protocol for PBMCs

**Figure 1:**
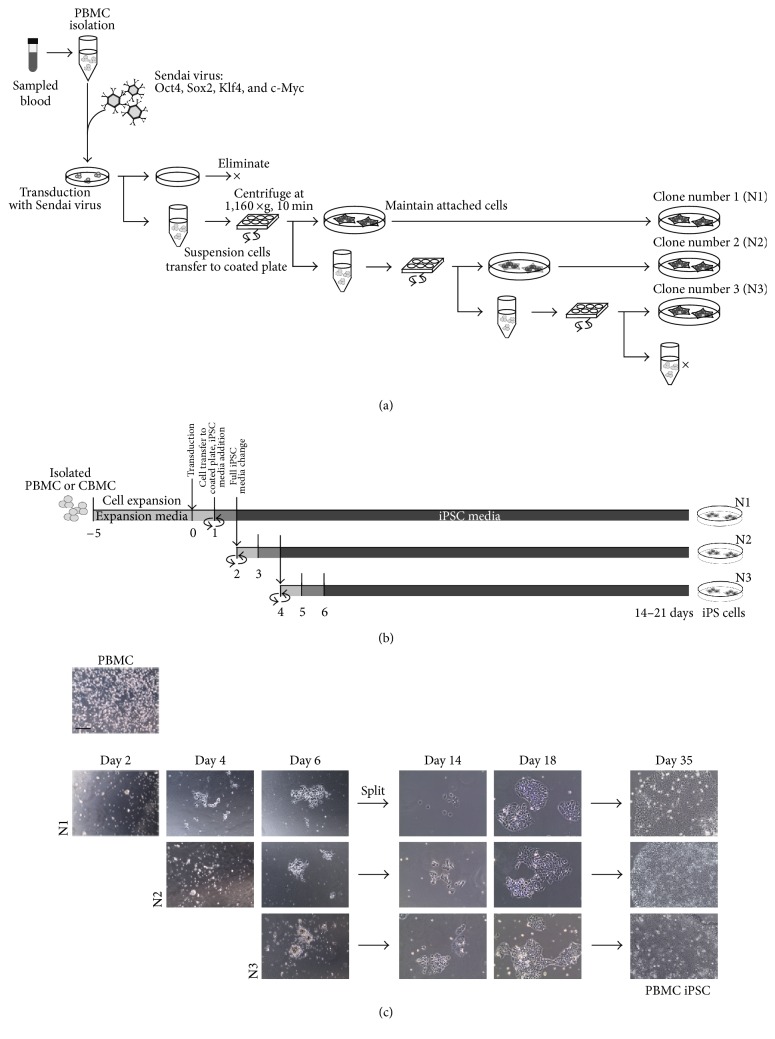
The scheme of the established protocol. (a) A detailed diagram of the designed protocol. (b) A simple scheme of the procedure over time based on medium type. (c) Brightfield image of generated cells. A greater number of colonies were obtained in a shorter amount of time. Scale bars: 200 *μ*m.

To generate iPSCs from PBMCs more effectively, we established a protocol including several additional steps (Figure 1(a)). To increase the initial adherence of transduced cells, we settled the cells using centrifugation. We repeatedly collected the nonadherent cells and replated them onto a new vitronectin-coated dish to increase the number of attached cells. Eventually, we were able to obtain at least three healthy wells of generated iPSC clones by repeated centrifugation: N1, N2, and N3. However, the amount of attached cells decreased after plating to the third well. A diagram of the protocol based on centrifugation and media type is shown in Figure 1(b). According to our protocol, PBMCs isolated from blood were incubated in expansion medium for 5 days. After expansion and stabilization in the expansion media, the cells were transfected with Yamanaka factors by Sendai viral vectors. The day after the infection, cells were transferred onto a vitronectin-coated plate and centrifuged to improve the cell attachment. Transduced PBMCs formed iPSC-like colonies 6 days after transduction. iPSC colonies with clean boundaries were obtained 18 days after infection. All three serially plated clones successfully reprogrammed into large iPSC colonies (Figure 1(c)). We simply confirmed that our protocol with serial plating by centrifugation improved the yield of iPSC colonies.

### 3.2. Serial Plating by Centrifugation Induces Efficient iPSC Reprogramming from PBMCs

**Figure 2:**
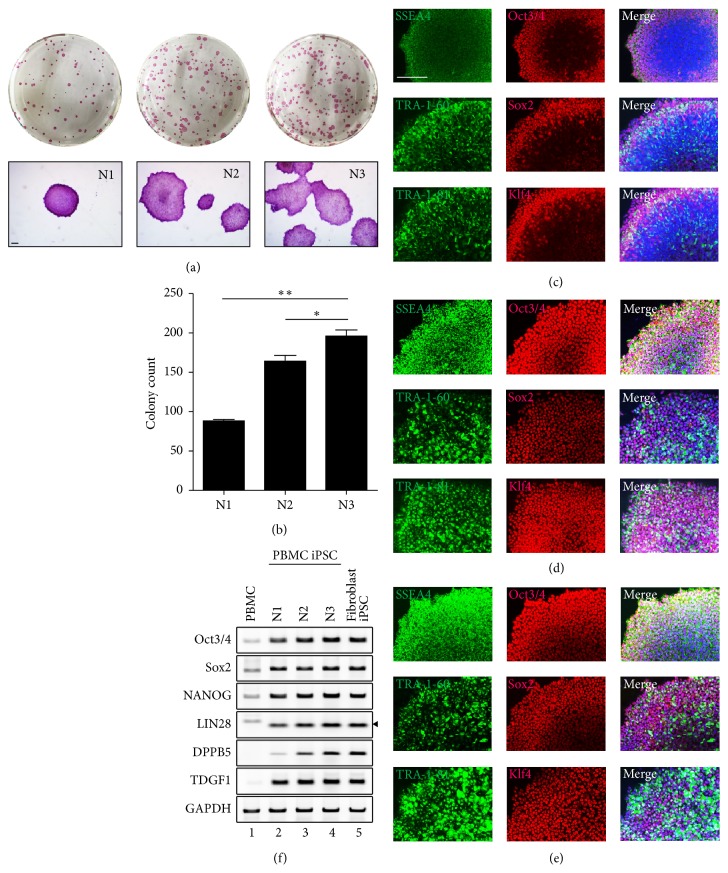
Stemness characterization of iPSCs generated by the established protocol. (a) Brightfield image of AP-stained clones. The N3 clone showed the highest number of colonies after 7 days. (b) Colony count of the AP-stained cells in Figure 2(a). Immunocytochemical staining of N1 (c), N2 (d), and N3 (e). N3 showed a slightly higher expression of pluripotency markers than N1 and N2. (^*∗*^ *P* < 0.05; ^*∗∗*^ *P* < 0.01). (f) Gene expression of pluripotency markers in each clone was confirmed by RT-PCR. Cells were compared to raw PBMCs and fibroblast-derived iPSCs. Scale bars: 200 *μ*m.

To confirm that our newly established protocol induces true pluripotency, several assays were performed (Figure 2). We obtained three wells of iPSC clones, N1, N2, and N3, from one well of transduced PBMCs through three rounds of serial plating with centrifugation (Figure 2(a)). We compared the number and size of iPSC colonies between N1, N2, and N3 using alkaline phosphatase (AP) staining (Figure 2(a)). N2 and N3 colony numbers were higher than that of N1 (Figure 2(b)). The size of the N3 colonies was greater than that of the N1 and N2 colonies; however, large size and number do not guarantee high pluripotency. Therefore, we measured the expression of the pluripotency markers SSEA4, Oct3/4, Tra-1-81, Sox2, Tra-1-60, and Klf4 in colonies acquired by serial plating (Figures 2(c), 2(d), and 2(e)). The expression of each marker was confirmed in all colonies. N2 and N3 showed higher expression of pluripotency makers than N1. However, there was no significant difference between all three clones. Additionally, we checked the marker expression by RT-PCR. We designed primers for four new markers, NANOG, LIN28, DPPB5, and TDGF1, and two originally confirmed markers, Oct3/4 and Sox2 (Figure 2(f)). Pluripotency markers, which were hardly expressed in PBMCs, were detected in all iPSC clones. The pluripotency marker expression of PBMC-derived iPSCs was similar to that of skin fibroblast-derived iPSCs (Figure 2(f), lanes 4 and 5). However, in the case of DPPB5, N3 showed higher expression than N1. These findings demonstrate that the second and third serially plated clones have higher pluripotent characteristics compared to the first clone. In conclusion, our protocol was able to generate a substantial number of iPSCs possessing the same level of pluripotency as fibroblast-derived iPSCs.

### 3.3. Multipotency of the iPSCs Generated by the Established Protocol

**Figure 3:**
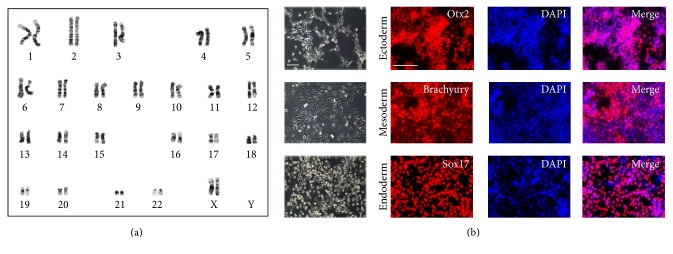
Further detailed characterization of N3. (a) High-resolution, G-banded karyotype of N3. Data show that the cell has a normal 44+XX chromosomal content. (b) Potential differentiation ability of N3 was confirmed using functional identification staining. Scale bars: 200 *μ*m.

To confirm that the generated iPSCs are genomically normal, we analyzed the karyotype of N3, the clone with the best quality (Figure 3(a)). Clone N3 showed a normal karyotype of 44+XX. Furthermore, to test the multipotent ability, we induced differentiation into ectoderm, mesoderm, and endoderm using the proper differentiation media (Figure 3(b)). Lineage commitment was evaluated by staining with anti-Sox17 antibody for endoderm, anti-Otx2 antibody for ectoderm, and anti-Brachyury antibody for mesoderm. Counterstaining was conducted with DAPI (blue). We observed the expression of the respective germ layer markers in each differentiated cell lineage. These results show that our protocol can generate iPSCs with no genomic mutation and that these iPSCs can differentiate into diverse lineages.

### 3.4. Generation of iPSCs from CBMCs Using the Established Protocol

**Figure 4:**
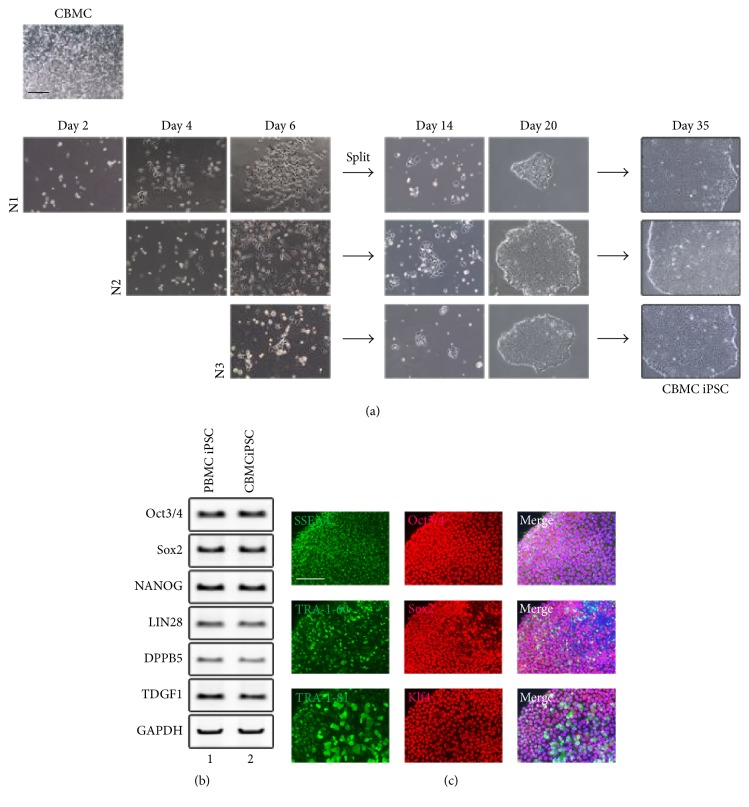
Generation of iPSCs from CBMCs with the established protocol. (a) Brightfield image of iPSCs generated from CBMCs. Colonies appeared in a similar time frame as PBMC iPSCs. (b) Gene expression of pluripotency markers in N3 from CBMC iPSCs showed similar expression to that of PBMC iPSC N3. (c) Immunocytochemical staining of CBMC iPSC clone N3. Scale bars: 200 *μ*m.

Since the protocol was designed for suspension cells, we tested whether cord blood mononuclear cells (CBMCs) could be reprogrammed into iPSCs using our established protocol. CBMCs were transduced and plated serially with centrifugation using the same protocol as PBMCs (Figure 4(a)). We observed iPSC-like colonies on day 14 after infection with Sendai virus. A substantial number of clear and round iPSC colonies appeared in the same time frame as in the PBMC differentiation. To check the pluripotency of CBMC-derived iPSCs, we measured the expression of pluripotency markers already tested in PBMC-derived iPSCs by RT-PCR and immunostaining (Figures 4(b) and 4(c)). The marker expression of CBMC-derived iPSC clone N3 was compared to that of the PBMC iPSC clone N3. The N3 clone of CBMC-derived iPSC showed similar marker expression as the PBMC-derived N3 clone. These data confirmed that a substantial number of pluripotent iPSC colonies could be obtained with our newly established protocol from CBMCs as well as PBMCs.

**Figure 5:**
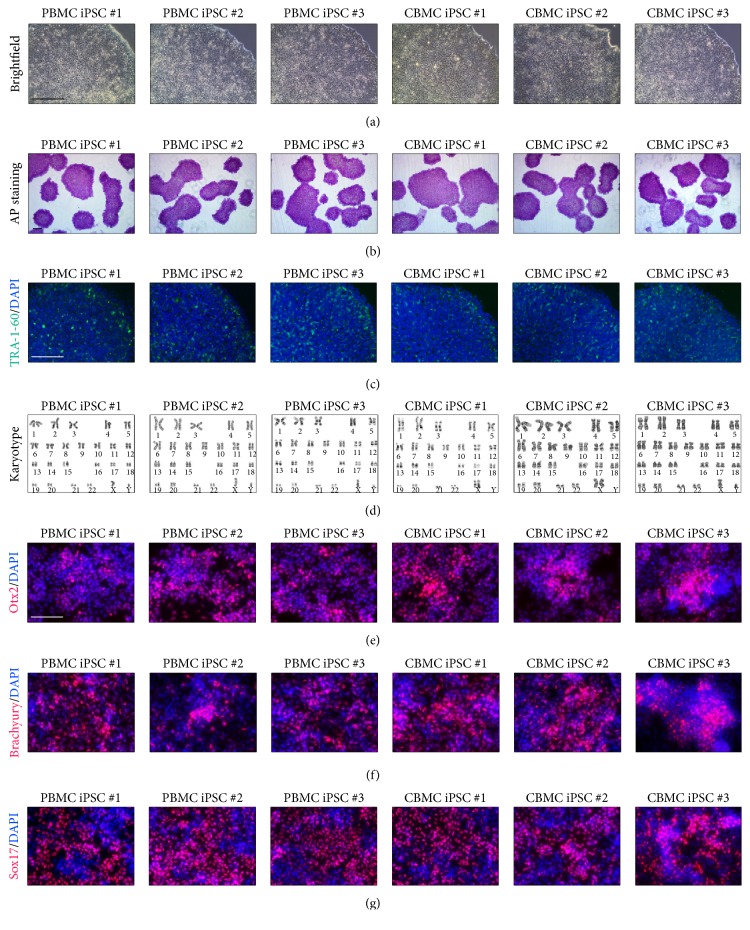
iPSCs generated using the established protocol. (a) Brightfield image of iPSCs generated from PBMCs and CBMCs. (b) Alkaline phosphatase staining results of generated iPSCs. (c) TRA-1-60 stained fluorescence image of iPSCs. (d) Karyotype results of generated iPSCs. All cell lines showed normal karyotype. (e) Ectoderm differentiation, (f) mesoderm differentiation, and (g) endoderm differentiation image of iPSCs. Scale bars: 200 *μ*m.

Using our protocol, more than 50 iPSC lines were developed. In Figure 5, we are showing the representative image of three PBMC-derived iPSCs and three CBMC-derived iPSCs. All six iPSC lines showed the common colony morphology (Figure 5(a)). Established iPSCs were positively stained against alkaline phosphatase and TRA-1-60 (Figures 5(b) and 5(c)). Cell lines maintained normal karyotype after reprogramming (Figure 5(d)). All cell lines differentiated into all three germ layers (Figures 5(e)–5(g)). With this protocol, our group was able to reprogram more than 50 patient-derived iPSCs from PBMCs and CBMCs.

## 4. Discussion

The first appearance of human ESCs in 1998 was thought to lead breakthroughs in cell therapy and other medical applications [5]. However, ethical issues were hard to overcome when using hESCs in research, prompting the search for an alternative cell source. In 2007, Yamanaka attempted to create ESC-like cells from mature somatic cells and succeeded in generating human induced pluripotent stem cells (hiPSCs) [1]. With similar characteristics as hESCs, but without any ethical debates, hiPSCs became a novel alternative in stem cell biology.

After the discovery of iPSCs, many scientists studied the reprogramming to iPSCs from various somatic cell types. Currently, various protocols exist, designed by numerous groups using diverse somatic cell types. The cell type generally used in the early days of iPSC reprogramming was fibroblasts. The reprogramming of fibroblasts was sufficiently efficient to obtain iPSCs; however, fibroblasts are difficult to obtain, due to the invasive procedure. In addition, fibroblasts require a mending process before being used for reprogramming. Unlike fibroblasts, peripheral blood cells can be easily obtained, avoiding surgical procedures [3]. Many groups attempted reprogramming using blood cells, but the reprogramming efficiency was low despite the laborious process.

The biggest difference between fibroblasts and PBMCs, in terms of reprogramming, is adherence. Simply, to reprogram PBMCs into iPSCs, the cells first need to transform into an attached form. Without attachment, iPSCs are not able to survive the reprogramming process. Moreover, according to the previous research of Pyle et al., hESC growth is known to be dependent on cell-cell interactions [13]. Usually, when reprogramming PBMCs, the biggest problem is the settlement of the reprogrammed cells. Reprogrammed cells proliferate while they are floating, and when they become large enough to sink, they start to attach by only gravity. However, during this process, these cells can be eliminated by daily media change. Therefore, even when they attach, the cell confluence is usually very low, which results in poor proliferation. Consequently, we decided to induce the settlement of the floating reprogrammed cells by centrifugation in order to improve the yield of attached reprogrammed cells. Therefore, we attempted to seed the cells serially onto a vitronectin-coated well by centrifugation to improve adherence and reprogramming. The settled cells were then able to expand into fully reprogrammed iPSCs. We obtained three plates or clones through serial plating. The second (N2) and third (N3) serially plated cells yielded bigger colonies than the cells from the first plating (N1) and showed a higher number of colonies as well. We were able to know that the numbers of attached reprogrammed cells increased through the serial plating process. However, serial plating was effective until N3, but attachment decreased afterwards. The iPSCs generated by our newly established protocol showed high expression of pluripotency markers and were able to differentiate into each of the three germ layers. In addition, the protocol was applicable not only to PBMCs but also to CBMCs. Also, our newly established protocol was useful when reprogramming cells from rare blood samples or from a small amount of blood cells (<5 mL).

iPSCs have great potential as therapeutic cells. Nonetheless, several safety issues concern us. Unlike ESCs, iPSCs have to be induced by several growth factors and feeders that contain various animal products; therefore, the use of such iPSCs in clinic presents the potential for contamination with animal pathogens [14]. However, in this study, our protocol used only defined chemicals and animal-free, xeno-free proteins for reprogramming. Also, integration by viral vectors was removable through early subcloning process (data not shown). Therefore, iPSCs made by our protocol can be used in clinical uses. However, the use of nonviral materials for reprogramming shall be needed to be completely safe for clinical trials, and our group is planning to apply in the future. We are looking forward to developing a standard operating procedure for the generation of clinical-grade iPSCs.

In summary, we propose a newly established protocol that improves the reprogramming efficiency of PBMC-derived iPSCs. The pluripotency of the PBMC-derived iPSCs generated by our method was proven by several methods. Moreover, we confirmed that our protocol was applicable to CBMCs. The iPSCs derived using our protocol can be used in therapeutic cell research, drug screening, and disease modeling. In addition, by inducing reprogramming with xeno-free, animal-free materials, our protocol can be adapted to produce cells for clinical use.

## 5. Conclusions

We propose a protocol that improves the reprogramming efficiency of PBMC-derived iPSCs. The pluripotency of the PBMC-derived iPSCs generated by our method was proven by several assays. Moreover, we confirmed that our protocol was applicable to CBMCs. The iPSCs derived using our protocol can be used in therapeutic cell research, drug screening, and disease modeling. In addition, by inducing reprogramming with xeno-free, animal-free materials, our protocol can be adapted to produce stem cells for clinical uses as regenerative medicine.

## Figures and Tables

**Table 1:** 

Target name	Direction	Primer sequence	Size
Oct3/4	Forward	ACCCCTGGTGCCGTGAA	190
	Reverse	GGCTGAATACCTTCCCAAATA	

Sox2	Forward	CAGCGCATGGACAGTTAC	321
	Reverse	GGAGTGGGAGGAAGAGGT	

NANOG	Forward	AAAGGCAAACAACCCACT	270
	Reverse	GCTATTCTTCGGCCAGTT	

LIN28	Forward	GTTCGGCTTCCTGTCCAT	122
	Reverse	CTGCCTCACCCTCCTTCA	

DPPB5	Forward	CGGCTGCTGAAAGCCATTTT	215
	Reverse	AGTTTGAGCATCCCTCGCTC	

TDGF1	Forward	TCCTTCTACGGACGGAACTG	140
	Reverse	AGAAATGCCTGAGGAAAGCA	

GAPDH	Forward	GAATGGGCAGCCGTTAGGAA	414
	Reverse	GACTCCACGACGTACTCAGC	
